# Supplementary material for: Combining passive acoustic data from a towed hydrophone array with visual line transect data to estimate abundance and availability bias of sperm whales (Physeter macrocephalus)
Source: PeerJ. 2023 Sep 21;11:e15850. doi: 10.7717/peerj.15850 (PMC10518167; doi:10.7717/peerj.15850)
Supplement: Supplemental Information 1 [file peerj-11-15850-s001.docx]

**Appendix S1: List of Challenges**

In this appendix we outline the numerous challenges we encountered when trying to combine these two types of data. This list is not intended to be collectively exhausted but provides a list of the issues we encountered.

1. *Duplicates*: The primary issue to consider when combining VLT with PAM data collected simultaneously is adjusting for animals that are observed by both platforms. This is not a problem when the platforms are not operating simultaneously.
2. *Repeat Divers*: Because animals are not individually marked in the PAM data but only identified by clicking, there is a possibility that two separate clicking events could represent the same individual. The probability of detecting an individual animal across two different dive cycles is a direct function of the size of the observation window (which needs to be defined *a priori*) for the towed array. For sperm whales, our data suggest this probability is relatively low for small time windows (<30 min), but not necessarily negligible.
3. *Depth*: Current software typically calculates an individual’s location from the trackline in two dimensions. Techniques for 3-D localization exist but require further post-processing of the data and in-situ knowledge of the depth of the hydrophone array which may not always be known. Therefore, the 2-D distance to a vocalizing animal represents a slant distance between the acoustic array to the whale at depth, in contrast to the perpendicular distance across the ocean surface between the array and animal, as is commonly used in distance sampling techniques (Marques et al., 2013, DeAngelis et al. 2017). The degree of bias is a direct function of the ratio of the depth of the vocalizing animal to the slant distance to that animal. For sperm whales this source of error is considered to be minimal (Barlow and Taylor 2005).
4. *Silent States*: For animals to be detected by the towed array they need to be vocalizing. There are a number of behavioral states where animals may be below the surface but in a non-clicking state and therefore not observable to either platform (Isojunno and Miller, 2015). For sperm whales, their clicking state while foraging is fairly regular, but they are initially silent for a short period at the beginning of a dive and enter a prolonged silent state when surfacing (Watwood et al., 2006). As this study is using only clicks from the foraging state, clicks emitted while in a social state (codas, slow clicks) are also considered “silent”. However, social clicks can be processed and included in future analyses (see Barkley et al. 2022 for an example).
5. *Blind spots*: The hull of the ship creates a blind spot for the passive acoustic array where whales clicking directly forward of the array up to some depth cannot be detected. Vocalizing animals that are closer to the trackline and/or at a far forward distance from the array and at the start of their dive (shallower) are more likely to be in the blind spot for an extended period. The size of the blind spot can vary depending on the ship, length of the tow cable, and the sound propagation properties in a given location (e.g. presence/absence of surface sound channels).
6. *Ambiguous click trains*- Sperm whales often dive and click within acoustic proximity of other clicking sperm whales, thus the towed array records clicks from multiple individuals simultaneously. Depending on the location of the clicking whales relative to the ship, these bearings can often be only a few degrees apart, causing click trains from separate individuals to overlap in bearing and become ambiguous (see Figure S2 in Appendix S2). In such a case, it is not possible to distinguish between individuals and only a portion of the recorded click train can be assigned to a specific individual.
7. *Annotating clicks*: Vocalizing sperm whales can produce large numbers of clicks (n= 1000-3000, Madsen et al. 2002) during their dive with inter-click intervals (ICIs) as short as 0.33 s. As a consequence, the ability of an analyst to manually assign every click to an individual is limited. This coupled with ambiguous click trains lead to most individuals having only portions of click trains assigned.
8. *Group size*: Unlike visual data where observers can enumerate the number of animals within a group, it is more challenging to determine the group size using PAM when there are overlapping click trains (e.g., beaked whales; Alcazar-Trevino et al 2021). We have assumed a click train reflects one individual sperm whale.

**References**

Alcázar-Treviño J, JohnsonM, Arranz P, Warren VE, Pérez-González CJ,Marques T, Madsen

PT, Aguilar de Soto N. 2021. Deep-diving beaked whales dive together but forage apart.

*Proceedings of the Royal Society B*, 288:20201905. <https://doi.org/10.1098/rspb.2020.1905>

Barkley, Y. M., Sakai, T., Oleson, E. M. & Franklin, E. C. 2022. Examining distribution

patterns of foraging and non-foraging sperm whales in Hawaiian waters using visual and passive acoustic data. *Frontiers in Remote Sensing* **3**:940186. doi: 10.3389/frsen.2022.940186.

Barlow J, Taylor BL. 2005. Estimates of sperm whale abundance in the northeastern temperate pacific from a combined acoustic and visual survey. *Marine Mammal Science* 21. DOI: 10.1111/j.1748-7692.2005.tb01242.x.

DeAngelis AI, Valtierra R, van Parijs SM, Cholewiak D. 2017. Using multipath reflections to obtain dive depths of beaked whales from a towed hydrophone array. *The Journal of the Acoustical Society of America* 142:1078–1087. DOI: 10.1121/1.4998709.

Isojunno S, Miller PJO. 2015. Sperm whale response to tag boat presence: Biologically informed hidden state models quantify lost feeding opportunities. http://www.esajournals.org/doi/pdf/10.1890/ES14-00130.1. *Ecosphere* 6. DOI: 10.1890/ES14-00130.1.

Madsen PT, Wahlberg M, Møhl B. 2002. Male sperm whale (Physeter macrocephalus) acoustics in a high-latitude habitat: Implications for echolocation and communication. *Behavioral Ecology and Sociobiology* 53. DOI: 10.1007/s00265-002-0548-1.

Marques TA, Thomas L, Martin SW, Mellinger DK, Ward JA, Moretti DJ, Harris D, Tyack PL. 2013. Estimating animal population density using passive acoustics. *Biological Reviews* 88:287–309. DOI: 10.1111/brv.12001.

Watwood SL, Miller PJO, Johnson M, Madsen PT, Tyack PL. 2006. Deep-diving foraging behaviour of sperm whales (Physeter macrocephalus). *Journal of Animal Ecology* 75:814–825. DOI: 10.1111/j.1365-2656.2006.01101.x.
