# Supplementary material for: Combining passive acoustic data from a towed hydrophone array with visual line transect data to estimate abundance and availability bias of sperm whales (Physeter macrocephalus)
Source: PeerJ. 2023 Sep 21;11:e15850. doi: 10.7717/peerj.15850 (PMC10518167; doi:10.7717/peerj.15850)
Supplement: Supplemental Information 2 [file peerj-11-15850-s002.docx]

**Appendix S2: Processing and formatting passive acoustic data**

*Processing passive acoustic data*

All sperm whale passive acoustic data were post-processed using the PAMGuard1.12.05 (Gillespie et al., 2008) click detector. Click detector settings were set to pre-filter the data with a Butterworth bandpass filter from 2 to 15 kHz and a trigger threshold set at 12 dB from 3 to 11 kHz. Localizing was done using PAMGuard’s Target Motion Analysis (TMA) 2D Simplex Optimisation algorithm. As TMA provides two possible solutions due to the left/right ambiguity resulting from using 2 hydrophones, the side PAMGuard selected with the best goodness of fit was chosen. PAMGuard exports the perpendicular distance of the position to the ship when the position reaches abeam.

Once subsequent click trains were grouped into events, the event ID, latitude and longitude position of the event, and each click’s timestamp and received bearing angle were exported and formatted into Excel spreadsheets using Matlab scripts (https://conservationcoding.com/2017/09/10/the-new-PAMGuard-matlab-library/). There, the posdist Excel function (NMML) was used to calculate the radial distance from the event position, to the position of the ship at the time of the click. The timestamps on the ship’s GPS feed was matched to the timestamp of the annotated clicks using custom built Matlab scripts. The latitude and longitude of ship at the closest timestamp was used to calculate the radial distance of the whale’s position to the ship at the time of the click. The bearings of each click were extracted from PAMGuard’s binary files using Matlab scripts from the PAMGuard -Matlab library. Using the radial distance and bearing, a forward distance was calculated for each click.

*Ambiguous events*

Some events were unambiguous and contained a limited number of clicks such that every click in the event could be annotated (see Figure S1 as an example). In cases of <3 individuals clicking at the same time along similar bearings, we could discern whether all three were clicking or not. A combination of capture history and annotated clicks was done in these cases. Ambiguity arose from multiple animals clicking within relatively close proximity to each other such that their received clicks appeared on similar bearings. In instances of >3 individuals, the events became completely ambiguous and no capture history was generated after the last unambiguous time bin (See Figure S2). Note that the time scales in both S1 and S2 were in some cases larger than the analysis time window, which varied from 1-15 min to adequately group click trains to the individual whale level.


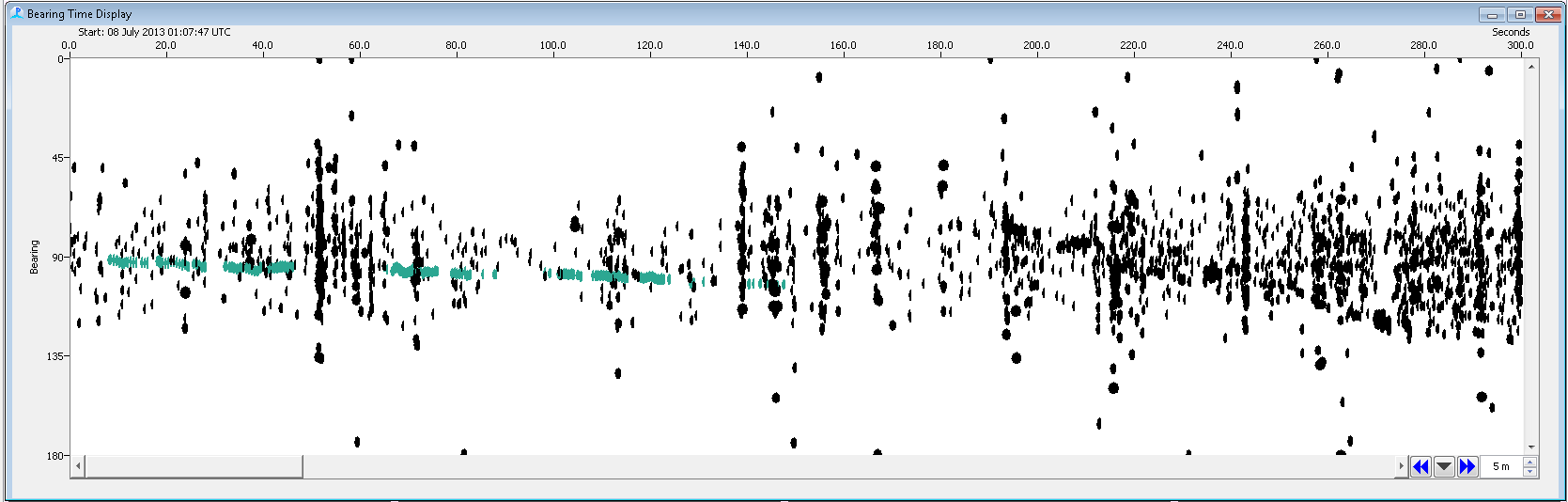


Figure S1: Unambiguous event (teal) where all the clicks were annotated in PAMGuard.


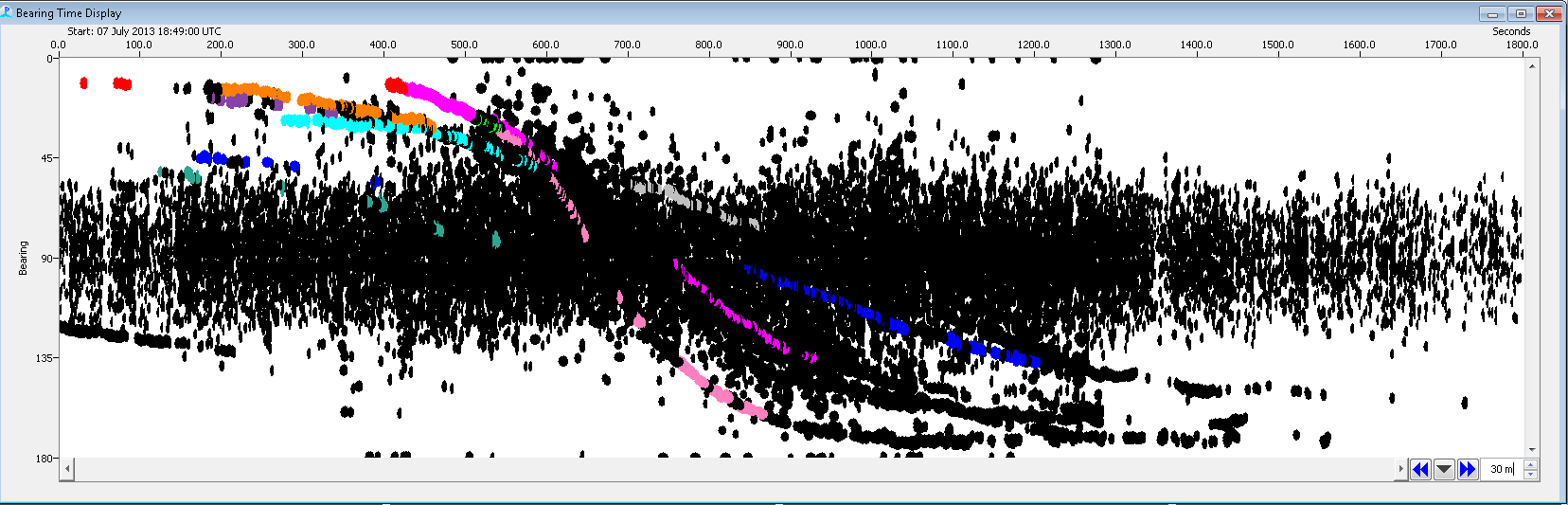


Figure S2: Ambiguous events where there is a lot of noise recorded on the array as well as many individuals clicking simultaneously. Only click trains during the greatest rate of bearing change were annotated per individual as there were many clicks recorded per individual. In this case, the bearing and distance information was collected from the annotated (colored) sections of events and capture histories were also generated for each event until points of complete ambiguity.
