# Supplementary material for: Combining passive acoustic data from a towed hydrophone array with visual line transect data to estimate abundance and availability bias of sperm whales (Physeter macrocephalus)
Source: PeerJ. 2023 Sep 21;11:e15850. doi: 10.7717/peerj.15850 (PMC10518167; doi:10.7717/peerj.15850)
Supplement: Supplemental Information 3 [file peerj-11-15850-s003.docx]

**Appendix S3: Description of the Zone of Overlap**

In this appendix we demonstrate the concept of the zone of overlap between the visual line transect (VLT) platform and the passive acoustic monitoring (PAM) platform and how silent states during the dive phase influence that zone. For foraging sperm whales there are generally two periods during the dive cycle when they are submerged but silent (Watwood et al. 2006), which are a brief period during the initial descent and a prolonged period during the ascent after foraging has ceased. Below we provide two schematics (Figures S3a & b) that represent slightly different scenarios for when a foraging whale is available for observation to both platforms and qualifies as a duplicate and when they are only observable by the PAM platform. In both scenarios, we assume the PAM platform has a greater range of detection than the VLT platform extending farther in front of the ship than the VLT platform and ranging behind the ship. The zone of detection for the VLT platform is represented by the red box and stops abeam of the ship. Therefore, as the ship approaches a whale it enters the zone of detection for the PAM array before it enters the zone of detection for the VLT and exits the zone of the detection for the VLT after the ship passes the whale.

In the first diagram (Figure S3a) there is a delay of one distance bin between when a whale initiates a dive and when it begins clicking (i. e., transitions to the foraging phase). Additionally, there is a gap of five bins between when a whale begins its silent ascent to the surface (i. e., transitions out of the foraging phase) and when it reaches the surface. In this scenario whale A initiates a dive outside of the zone of overlap with the VLT platform but transitions to the foraging phase within the zone of overlap. This whale does not qualify as a duplicate because it was never observable by the VLT platform. Similarly, whale B enters the zone of overlap in the foraging phase where it is observable to the PAM platform and also transitions to the surfacing phase within the zone of overlap but does not reach the surface until it is outside of the zone of overlap and therefore, is also never observable to the VLT platform.

In the second diagram (Figure S3b) whale A is inside the zone of overlap and observable by the VLT platform before it initiates its dive and then starts clicking and becomes observable to the PAM platform. Conversely, whale B transitions out of the foraging state after bin 3 and becomes observable to the VLT platform within the zone of overlap. Whales that are still in the foraging state after Bin 2 would not qualify as duplicates because they would not be available to the VLT team by the time they surface. Therefore, in this scenario only the first three bins should be included in the calculation of $S_{T}$ because any whale that transitions to the surfacing phase beyond bin 3 would surface outside the zone of overlap. Conversely, bins 1 and 2 should not be included in the calculation of $F_{T}$ because whales that transition to the foraging phase in these two bins would have initiated their dives outside the zone of overlap.


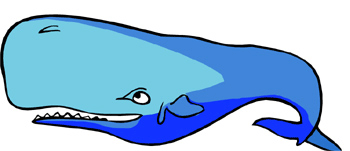


**Bin 1**

**Bin 2**

**Bin 3**

**Bin 4**

**Bin 5**


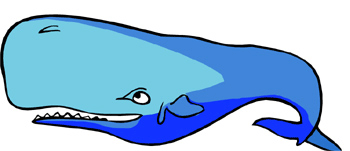


**Bin 6**

**Bin 7**

**Bin 8**

**Bin 9**

**Bin 9**

**Bin 10 10**

**Bin 11**

**A**

**B**

**a)**

**b)**


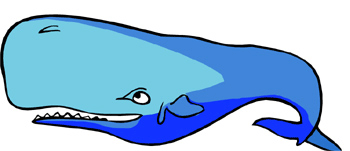


**Bin 1**

**Bin 2**

**Bin 3**

**Bin 4**

**Bin 5**


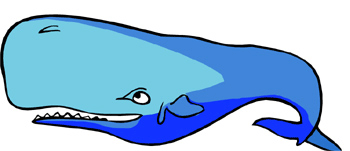


**Bin 6**

**Bin 7**

**Bin 8**

**Bin 9**

**Bin 10**

**Bin 11**

**A**

**B**

Figure S3a & b: Schematics representing when whales are not available to both platforms (a) and when they are available to both platforms (b). The red box represents the detection zone for the VLT platform which is also the zone of overlap between the VLT platform and the PAM platform. Black lines represent whales transitioning from the surface to the foraging sate (Whale A in both figures) or from the foraging state to the surface (Whale B in both figures). The yellow dotted lines represent periods during the dive cycle when a whale is clicking and observable to the PAM platform.
