# Supplementary material for: Combining passive acoustic data from a towed hydrophone array with visual line transect data to estimate abundance and availability bias of sperm whales (Physeter macrocephalus)
Source: PeerJ. 2023 Sep 21;11:e15850. doi: 10.7717/peerj.15850 (PMC10518167; doi:10.7717/peerj.15850)
Supplement: Supplemental Information 4 [file peerj-11-15850-s004.docx]

**Appendix S4:** **Description of Simulations**

*General Design*

For all simulations we defined the zone of detection for the PAM platform to have a maximum forward distance (Y) and maximum perpendicular distance (X) of 7 KM. We simulated a smaller zone of detection for the VLT platform where the zone of overlap had a maximum Y and a maximum X of 5 KM. The zone of overlap also had a minimum Y of 0 KM such that whales on the surface behind the ship (past 90°) are not observable to the VLT platform. The total abundance was set at 500 whales in all simulations. Each simulated whale started at a forward distance of 7 KM. We assigned a random perpendicular distance between 0 and 7 KM to each simulated whale. For convenience, we set the speed of the ship to 250 meters per minute and the width of the forward bins to 250 meters so that the ship transitions among bins at a rate of 1 distance bin per minute. We updated the simulation at one minute intervals for a total of 60 minutes where the ship moves 250 meters closer to the whale each minute until it passes the whale at which point the PAM platform continues to detect the whale until it is out of range or until the 60 minutes simulation time is up. For each simulated whale, X is fixed throughout the simulation.

We simulated diving behavior was based on digital tag information reported in Watwood et al. (2006) of sperm whales in the Atlantic Ocean. We randomly assigned a dive cycle length to each whale using a normal distribution with a mean of 55 minutes and a standard deviation of 2 where the dive cycle includes the surface phase. At the start of each simulation, we also randomly assigned each whale to a specific point in its dive cycle based on its total dive cycle length. Next, we divided the dive cycle into four distinct phases which included 1) a surface phase where the whale is resting at the surface, 2) a descent phase where the whale is actively diving, 3) a bottom phase where the whale is foraging at a constant depth and 4) an ascent phase where the whale is swimming to the surface (see Figure S4). The percentage of time in each dive phase was calculated from the average observed times in each of these phases reported by Watwood et al. (2006) (see Figure S4 for percentages). Based on these percentages and the total dive cycle length we calculated the amount of time in each dive phase. The maximum depth was based on the total time in the descent phase and the average descent rate reported in Watwood et al. (2006) (1.2 meters per second). Conversely, the ascent rate was calculated using the maximum depth and the total amount of time in the ascent phase. Using the maximum depth, descent rate and ascent rate we calculate the depth of the simulated whale at the start of each simulation. For example, if a whale has been in its diving phase for 5 minutes at the start of the simulation, we multiply the descent rate by 5 minutes to determine how deep it currently is at the initial time point. We then update its depth at each iteration of the simulation.

To replicate the error in position that is given by PAMGuard we first calculated the slant range based on its current three dimensional position using the equation

$${SR}_{it}=X_{i}^{2}+Y_{it}^{2}+Z_{it}^{2}$$

where *X_i_* represents the perpendicular position of whale *i*, *Y_it_* is the current forward position of whale *i* at time *t*, *Z_it_* is the current depth of whale *i* at time *t* and *SR_it_* is the current slant range of whale *i* at time *t*. We calculated the current bearing using the true *X* and *Y* position at time *t*. Next, we assume a horizontal position by treating the slant range as a radial distance and re-calculated *X* and *Y* based on this presumed radial distance and the current bearing.

*Detection*

To simulate detection by the PAM array we used a logistic model where detection was a function of true radial distance. From this simulated data we constructed the capture history matrix for the CMR sub model. For the VLT platform we used a half-normal detection function. For the Hybrid model we randomly took a subset (n=35) from the complete capture history matrix as input to the CMR component of this model. The Hybrid model also requires an input dataset of perpendicular distances from the PAM platform for the distance sampling submodel. Similar to the VLT data we also simulated this input data set from a half normal based on the perpendicular distances.

**17% 6% 11% 50% 16%**

**DEPTH (meters)**


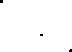


Silent

Ascent

Phase

Clicking

Foraging

Phase

Silent

Diving

Phase

Clicking

Diving

Phase

Surface

Phase

Max Time

0

**TIME (minutes)**

Figure S4: Diagram of the five dive phases used in simulations to simulate the dive cycle of sperm whales. The black dotted lines represents the acoustically active phases of the dive cycle where whales can be detected by the acoustic array.
