# Supplementary material for: Combining passive acoustic data from a towed hydrophone array with visual line transect data to estimate abundance and availability bias of sperm whales (Physeter macrocephalus)
Source: PeerJ. 2023 Sep 21;11:e15850. doi: 10.7717/peerj.15850 (PMC10518167; doi:10.7717/peerj.15850)
Supplement: Supplemental Information 5 [file peerj-11-15850-s005.docx]

**Appendix S5: Description of sperm whale analysis with *Hybrid Method***

To analyze the sperm whale dataset it was necessary to make several decisions with respect to the input data and analysis which included:

1. Parametrization of the distance sampling (DS) analysis for both visual line transect (VLT) and passive acoustic monitoring (PAM) data
2. Setting up a capture history matrix for the capture-mark-recapture (CMR) analysis
3. Deciding on the size of the zone of overlap

Below we discuss each step in detail.

*DS Analysis*

To analyze the VLT data we implemented the Bayesian mark-recapture distance sampling (MRDS) method described in Sigourney et al. (2020). The top model was determined from a previous analysis of this dataset reported in Palka et al. (2017). For the DS model we used a hazard rate function without detection covariates and a truncation distance of 7.6 KM. The MR model included an interaction between observer team and distance. We analyzed the passive acoustic data with a hazard rate function and the same truncation distance as the visual data. We did not include an MR component to model acoustic data and therefore assumed that *p(0)*=1.

*Capture history matrix*

We processed a total of 72 click trains that could be used in the CMR analysis of the *Hybrid Method*. Because the click trains included some partially annotated click trains that we divided into one minute time intervals (see Appendix S1) we set the width of each distance bin to 309 meters which approximates a time interval of one minute given the speed of the ship. Initially, we set the first bin at 10 KM and the last bin at a forward distance of -10 KM for a total of 64 bins. This resulted in a sparse capture history matrix. To analyze the data we truncated the matrix starting at bin 18 and ending at bin 48 such that the first bin started at a forward distance of approximately 4.8 KM and ended at a forward distance of approximately-3.6 KM.

*Zone of Overlap*

To fully implement the *Hybrid Method* there are several user-defined inputs that need to be established in order to estimate the parameters that ultimately define the total number of duplicates which include 1) specifying *Y_Min_* and *Y_Max_* to estimate $n_{B}^{(H)}$ 2) calculating $\bar{f}^{(H)}$ and $\bar{s}^{(H)}$ and 3) specifying ${Zone}_{F}$ and ${Zone}_{S}$ for calculating $F_{T}^{(H)}$ and $S_{T}^{(H)}$, respectively.

For calculating $n_{B}^{(H)}$, *Y_Max_* and *Y_Min_* should be chosen such that the total distance between *Y_Max_* and *Y_Min_* is the same distance as the assumed window of overlap between the VLT platform and PAM platform. Therefore, we chose *Y_Max_* to be the first bin and *Y_Min_* to be bin 25 and $n_{B}^{(H)}$ as

$$n_{B}^{(H)}=\sum_{i=1}^{M} I\left\{ \sum_{j=1}^{j=25} z_{ij}>0 \right\}$$

where 25 bins is approximately equal to 7.6 KM which is the size of the assumed zone of overlap.

To calculate $\bar{f}^{(H)}$ and $\bar{s}^{(H)}$, the user also needs to define what bins to average over. Because capture histories beyond 90° were sparse we chose to be in the middle of the capture history matrix where the numbers entering the dive phase and the surfacing phase appeared to be constant. To do so, we averaged from bin 7 to bin 16 such that the total number of bins was equal to 10 and came from the middle of the data matrix. Therefore, we estimated $\bar{f}^{(H)}$ and $\bar{s}^{(H)}$ as

$\bar{f}^{(H)}=\frac{\sum_{j=7}^{16} f_{j}^{(H)}}{10}$,

and

$\bar{s}^{(H)}=\frac{\sum_{j=7}^{16} s_{j}^{(H)}}{10}$,

respectively.

Finally, when specifying the size of ${Zone}_{F}$ and ${Zone}_{S}$ where duplicates can occur, it is necessary to consider over what part of the dive cycle animals are vocalizing and therefore available to be detected by the acoustic array. We assumed because sperm whales are silent during a prolonged ascent phase and not immediately available to the VLT platform such that whales transitioning from the foraging state to the surfacing state ($\boldsymbol{S}_{\boldsymbol{T}}^{\boldsymbol{(H)}}$) would only contribute to a small fraction of the number of duplicates. Accordingly, we set ${Zone}_{S}$ to 10 such that whales that transition to the surface state within the first 10 distance bins are considered observable by both platforms, but whales transitioning beyond this zone have a low probability of being observable to the VLT platform. Similarly, there is also an initial silent period at the beginning of the dive phase where animals are not vocalizing, therefore, we set ${Zone}_{F}$ to 22 as the first three minutes of the dive animals are not immediately available for detection to the towed array because they are not vocalizing.
