# Supplementary material for: Combining passive acoustic data from a towed hydrophone array with visual line transect data to estimate abundance and availability bias of sperm whales (Physeter macrocephalus)
Source: PeerJ. 2023 Sep 21;11:e15850. doi: 10.7717/peerj.15850 (PMC10518167; doi:10.7717/peerj.15850)
Supplement: Supplemental Information 6 — Posterior summaries of parameters from the analysis of the sperm whale data with the Hybrid Method. Gelman-Rubin statistics (G–R) and effective samples sizes (Eff) are included. [file peerj-11-15850-s006.docx]

Table S1: **Posterior summaries of parameters.** Posterior summaries of parameters from the analysis of the sperm whale data with the *Hybrid Method*. Gelman-Rubin statistics (G-R) and effective samples sizes (Eff) are included.

| Parameter | Median | SD | 2.50% | 97.50% | G-R | Eff |
| --- | --- | --- | --- | --- | --- | --- |
| F[1] | 25.00 | 6.24 | 15.00 | 39.00 | 1.00 | 452.61 |
| F[2] | 11.00 | 4.60 | 3.00 | 21.00 | 1.00 | 3512.10 |
| F[3] | 3.00 | 2.73 | 0.00 | 10.00 | 1.00 | 4503.40 |
| F[4] | 8.00 | 3.41 | 3.00 | 16.00 | 1.00 | 4095.60 |
| F[5] | 13.00 | 3.95 | 6.00 | 22.00 | 1.00 | 2876.36 |
| F[6] | 14.00 | 3.90 | 8.00 | 23.00 | 1.00 | 3290.77 |
| F[7] | 4.00 | 2.63 | 0.00 | 11.00 | 1.00 | 5370.14 |
| F[8] | 5.00 | 2.31 | 2.00 | 11.00 | 1.00 | 5355.60 |
| F[9] | 10.00 | 2.71 | 6.00 | 16.00 | 1.00 | 2326.50 |
| F[10] | 3.00 | 1.85 | 1.00 | 8.00 | 1.00 | 5430.58 |
| F[11] | 5.00 | 1.91 | 2.00 | 9.00 | 1.00 | 3107.12 |
| F[12] | 2.00 | 1.29 | 0.00 | 5.00 | 1.00 | 4822.22 |
| F[13] | 2.00 | 1.21 | 0.00 | 5.00 | 1.00 | 3681.98 |
| F[14] | 0.00 | 0.91 | 0.00 | 3.00 | 1.00 | 4397.31 |
| F[15] | 9.00 | 2.42 | 6.00 | 15.00 | 1.01 | 684.66 |
| F[16] | 5.00 | 2.06 | 2.00 | 10.00 | 1.00 | 1581.93 |
| F[17] | 1.00 | 1.22 | 0.00 | 4.00 | 1.00 | 5445.13 |
| F[18] | 1.00 | 0.98 | 0.00 | 4.00 | 1.00 | 3903.81 |
| F[19] | 1.00 | 0.69 | 0.00 | 3.00 | 1.00 | 2360.39 |
| F[20] | 0.00 | 0.31 | 0.00 | 1.00 | 1.00 | 3242.41 |
| F[21] | 0.00 | 0.21 | 0.00 | 1.00 | 1.00 | 4172.31 |
| F[22] | 0.00 | 0.14 | 0.00 | 0.00 | 1.00 | 5271.73 |
| F[23] | 0.00 | 0.11 | 0.00 | 0.00 | 1.00 | 4698.41 |
| F[24] | 0.00 | 0.08 | 0.00 | 0.00 | 1.01 | 4345.98 |
| F[25] | 0.00 | 0.05 | 0.00 | 0.00 | 1.02 | 6621.66 |
| F[26] | 0.00 | 0.04 | 0.00 | 0.00 | 1.01 | 3790.16 |
| F[27] | 0.00 | 0.03 | 0.00 | 0.00 | 1.21 | 11413.65 |
| F[28] | 0.00 | 0.02 | 0.00 | 0.00 | 1.29 | 1931.01 |
| F[29] | 0.00 | 0.02 | 0.00 | 0.00 | 1.29 | 4810.47 |
| F[30] | 0.00 | 0.01 | 0.00 | 0.00 | 1.29 | 10000.00 |
| F[31] | 0.00 | 0.01 | 0.00 | 0.00 | 1.29 | 2694.55 |
| N[1] | 25.00 | 6.24 | 15.00 | 39.00 | 1.00 | 452.61 |
| N[2] | 33.00 | 5.29 | 24.00 | 44.00 | 1.00 | 357.66 |
| N[3] | 33.00 | 4.74 | 25.00 | 43.00 | 1.00 | 492.21 |
| N[4] | 39.00 | 4.78 | 30.00 | 49.00 | 1.00 | 633.92 |
| N[5] | 49.00 | 4.75 | 40.00 | 59.00 | 1.00 | 1039.90 |
| N[6] | 60.00 | 4.88 | 51.00 | 70.00 | 1.01 | 1006.37 |
| N[7] | 61.00 | 4.56 | 52.00 | 70.00 | 1.01 | 649.69 |
| N[8] | 61.00 | 4.49 | 53.00 | 70.00 | 1.01 | 594.08 |
| N[9] | 67.00 | 4.54 | 59.00 | 76.00 | 1.01 | 556.46 |
| N[10] | 67.00 | 4.48 | 59.00 | 76.00 | 1.00 | 476.94 |
| N[11] | 69.00 | 4.42 | 61.00 | 78.00 | 1.00 | 349.48 |
| N[12] | 66.00 | 4.36 | 58.00 | 75.00 | 1.00 | 348.79 |
| N[13] | 65.00 | 4.28 | 57.00 | 73.00 | 1.00 | 357.53 |
| N[14] | 61.00 | 4.17 | 54.00 | 70.00 | 1.00 | 248.94 |
| N[15] | 65.00 | 4.52 | 57.00 | 74.00 | 1.01 | 217.63 |
| N[16] | 65.00 | 4.49 | 58.00 | 75.00 | 1.01 | 155.47 |
| N[17] | 60.00 | 4.31 | 52.00 | 69.00 | 1.01 | 137.90 |
| N[18] | 58.00 | 4.11 | 51.00 | 67.00 | 1.01 | 133.81 |
| N[19] | 54.00 | 3.95 | 47.00 | 62.00 | 1.01 | 131.03 |
| N[20] | 47.00 | 3.77 | 40.00 | 55.00 | 1.00 | 141.26 |
| N[21] | 42.00 | 3.55 | 37.00 | 50.00 | 1.00 | 122.56 |
| N[22] | 38.00 | 3.36 | 32.00 | 45.00 | 1.00 | 132.99 |
| N[23] | 32.00 | 3.19 | 27.00 | 39.00 | 1.00 | 136.76 |
| N[24] | 29.00 | 3.00 | 24.00 | 36.00 | 1.00 | 132.50 |
| N[25] | 25.00 | 2.89 | 20.00 | 31.00 | 1.00 | 141.56 |
| N[26] | 20.00 | 2.78 | 16.00 | 26.00 | 1.00 | 142.53 |
| N[27] | 15.00 | 2.69 | 11.00 | 22.00 | 1.00 | 166.60 |
| N[28] | 14.00 | 2.52 | 10.00 | 20.00 | 1.00 | 184.70 |
| N[29] | 11.00 | 2.50 | 7.00 | 17.00 | 1.00 | 197.23 |
| N[30] | 10.00 | 2.35 | 6.00 | 16.00 | 1.00 | 205.48 |
| N[31] | 9.00 | 2.22 | 6.00 | 15.00 | 1.00 | 238.08 |
| N.total | 425.58 | 123.54 | 332.37 | 813.94 | 1.00 | 2323.02 |
| N_Above | 305.88 | 116.28 | 216.38 | 668.24 | 1.00 | 2415.42 |
| N_Above_total | 515.82 | 299.28 | 293.90 | 1397.52 | 1.00 | 4290.68 |
| N_Below | 288.93 | 23.56 | 248.94 | 334.72 | 1.00 | 13153.61 |
| Nsuper | 128.00 | 0.06 | 128.00 | 128.00 | 1.08 | 2881.14 |
| P0_Above | 0.80 | 0.08 | 0.61 | 0.92 | 1.00 | 2374.04 |
| S[2] | 3.00 | 2.00 | 0.00 | 8.00 | 1.00 | 697.33 |
| S[3] | 3.00 | 1.92 | 0.00 | 7.00 | 1.00 | 764.04 |
| S[4] | 3.00 | 1.73 | 0.00 | 7.00 | 1.00 | 1172.65 |
| S[5] | 3.00 | 1.81 | 0.00 | 7.00 | 1.00 | 1489.93 |
| S[6] | 3.00 | 1.95 | 0.00 | 8.00 | 1.00 | 1755.52 |
| S[7] | 4.00 | 2.07 | 1.00 | 9.00 | 1.00 | 1628.40 |
| S[8] | 4.00 | 1.93 | 1.00 | 9.00 | 1.00 | 2719.50 |
| S[9] | 4.00 | 1.86 | 1.00 | 8.00 | 1.00 | 3743.85 |
| S[10] | 3.00 | 1.88 | 0.00 | 8.00 | 1.00 | 3775.31 |
| S[11] | 3.00 | 1.72 | 0.00 | 7.00 | 1.00 | 4383.36 |
| S[12] | 5.00 | 1.68 | 2.00 | 8.00 | 1.00 | 6393.98 |
| S[13] | 3.00 | 1.56 | 1.00 | 7.00 | 1.00 | 6588.88 |
| S[14] | 4.00 | 1.50 | 2.00 | 7.00 | 1.00 | 5460.50 |
| S[15] | 6.00 | 1.47 | 3.00 | 9.00 | 1.00 | 5950.54 |
| S[16] | 5.00 | 1.53 | 3.00 | 9.00 | 1.00 | 3291.78 |
| S[17] | 7.00 | 1.49 | 5.00 | 10.00 | 1.00 | 2240.20 |
| S[18] | 3.00 | 1.37 | 1.00 | 6.00 | 1.00 | 2841.28 |
| S[19] | 5.00 | 1.28 | 4.00 | 8.00 | 1.00 | 2501.12 |
| S[20] | 7.00 | 1.30 | 5.00 | 10.00 | 1.00 | 3118.80 |
| S[21] | 4.00 | 1.22 | 2.00 | 7.00 | 1.00 | 3235.24 |
| S[22] | 5.00 | 1.13 | 3.00 | 7.00 | 1.00 | 3286.24 |
| S[23] | 6.00 | 1.16 | 4.00 | 8.00 | 1.00 | 4738.95 |
| S[24] | 3.00 | 1.10 | 1.00 | 5.00 | 1.00 | 6696.15 |
| S[25] | 4.00 | 1.09 | 2.00 | 7.00 | 1.00 | 5988.95 |
| S[26] | 5.00 | 1.16 | 3.00 | 7.00 | 1.00 | 7031.80 |
| S[27] | 4.00 | 1.16 | 2.00 | 7.00 | 1.00 | 7780.61 |
| S[28] | 2.00 | 0.99 | 0.00 | 4.00 | 1.00 | 6515.09 |
| S[29] | 3.00 | 1.02 | 1.00 | 5.00 | 1.00 | 7622.98 |
| S[30] | 1.00 | 0.86 | 0.00 | 3.00 | 1.00 | 5956.96 |
| S[31] | 0.00 | 0.67 | 0.00 | 2.00 | 1.00 | 3075.91 |
| availability | 0.70 | 0.06 | 0.59 | 0.84 | 1.00 | 1274.47 |
| b.df.0_Above | 2.49 | 1.01 | 0.25 | 4.03 | 1.00 | 2465.81 |
| b.df.0_Below | 3.14 | 0.36 | 2.44 | 3.88 | 1.00 | 9876.61 |
| b.dist | -0.78 | 0.05 | -0.88 | -0.69 | 1.00 | 774.82 |
| b.dist1_Above | -0.14 | 0.17 | -0.47 | 0.19 | 1.00 | 2612.34 |
| b.dist2_Above | -0.20 | 0.13 | -0.47 | 0.04 | 1.00 | 2680.16 |
| b.time | -0.01 | 0.02 | -0.04 | 0.02 | 1.00 | 707.17 |
| b0 | 5.43 | 0.29 | 4.89 | 6.04 | 1.00 | 1978.14 |
| b0.phi | 2.41 | 0.24 | 2.00 | 2.94 | 1.00 | 593.83 |
| b1.0_Above | 0.51 | 0.46 | -0.38 | 1.43 | 1.00 | 2841.19 |
| b2.0_Above | -0.15 | 0.35 | -0.86 | 0.53 | 1.00 | 2953.32 |
| b_Above | 1.70 | 0.81 | 0.56 | 3.55 | 1.00 | 3165.42 |
| b_Below | 2.57 | 0.51 | 1.71 | 3.70 | 1.00 | 10102.37 |
| duplicates | 186.80 | 21.77 | 150.79 | 235.54 | 1.01 | 292.74 |
| fit.pred | 76.01 | 11.04 | 55.79 | 98.98 | 1.00 | 4949.74 |
| fit.true | 69.98 | 4.19 | 62.34 | 78.67 | 1.00 | 3742.81 |
| gamma[1] | 0.11 | 0.05 | 0.03 | 0.23 | 1.00 | 3422.24 |
| gamma[2] | 0.04 | 0.04 | 0.00 | 0.13 | 1.00 | 4159.59 |
| gamma[3] | 0.10 | 0.05 | 0.03 | 0.22 | 1.00 | 4767.78 |
| gamma[4] | 0.17 | 0.06 | 0.07 | 0.31 | 1.00 | 3667.41 |
| gamma[5] | 0.23 | 0.07 | 0.10 | 0.38 | 1.00 | 3317.80 |
| gamma[6] | 0.09 | 0.06 | 0.01 | 0.25 | 1.00 | 5733.77 |
| gamma[7] | 0.12 | 0.06 | 0.03 | 0.27 | 1.00 | 5978.09 |
| gamma[8] | 0.25 | 0.08 | 0.11 | 0.43 | 1.00 | 3547.15 |
| gamma[9] | 0.12 | 0.08 | 0.02 | 0.31 | 1.00 | 6497.61 |
| gamma[10] | 0.18 | 0.09 | 0.05 | 0.39 | 1.00 | 4389.07 |
| gamma[11] | 0.10 | 0.08 | 0.01 | 0.31 | 1.00 | 7190.23 |
| gamma[12] | 0.11 | 0.08 | 0.01 | 0.32 | 1.00 | 6790.79 |
| gamma[13] | 0.05 | 0.07 | 0.00 | 0.25 | 1.00 | 6374.99 |
| gamma[14] | 0.50 | 0.14 | 0.24 | 0.76 | 1.00 | 8844.69 |
| gamma[15] | 0.59 | 0.18 | 0.22 | 0.89 | 1.00 | 6799.32 |
| gamma[16] | 0.36 | 0.23 | 0.02 | 0.86 | 1.00 | 8427.87 |
| gamma[17] | 0.47 | 0.25 | 0.04 | 0.93 | 1.00 | 9418.14 |
| gamma[18] | 0.69 | 0.24 | 0.13 | 0.99 | 1.00 | 11610.00 |
| gamma[19] | 0.50 | 0.29 | 0.03 | 0.97 | 1.00 | 16152.34 |
| gamma[20] | 0.49 | 0.29 | 0.02 | 0.98 | 1.00 | 15948.11 |
| gamma[21] | 0.50 | 0.29 | 0.02 | 0.98 | 1.00 | 18601.54 |
| gamma[22] | 0.50 | 0.29 | 0.02 | 0.97 | 1.00 | 18588.15 |
| gamma[23] | 0.50 | 0.29 | 0.02 | 0.98 | 1.00 | 18931.25 |
| gamma[24] | 0.50 | 0.29 | 0.02 | 0.97 | 1.00 | 19279.92 |
| gamma[25] | 0.50 | 0.29 | 0.03 | 0.98 | 1.00 | 18820.22 |
| gamma[26] | 0.50 | 0.29 | 0.03 | 0.97 | 1.00 | 19312.56 |
| gamma[27] | 0.50 | 0.29 | 0.02 | 0.97 | 1.00 | 18948.79 |
| gamma[28] | 0.50 | 0.29 | 0.02 | 0.98 | 1.00 | 19373.53 |
| gamma[29] | 0.50 | 0.29 | 0.03 | 0.97 | 1.00 | 18699.97 |
| gamma[30] | 0.50 | 0.29 | 0.03 | 0.98 | 1.00 | 18996.05 |
| gamma1 | 0.20 | 0.06 | 0.10 | 0.33 | 1.00 | 766.10 |
| mean.F | 1.27 | 0.21 | 0.93 | 1.73 | 1.01 | 198.94 |
| mean.S | 4.60 | 0.28 | 4.13 | 5.20 | 1.01 | 274.36 |
| mean.esw_Above | 3.71 | 0.82 | 1.69 | 4.96 | 1.00 | 2672.61 |
| mean.esw_Below | 4.11 | 0.31 | 3.52 | 4.76 | 1.00 | 13194.48 |
| p.F | 0.01 | 0.00 | 0.01 | 0.01 | 1.01 | 198.93 |
| p.S | 0.04 | 0.00 | 0.03 | 0.04 | 1.01 | 274.30 |
| totF | 19.00 | 3.15 | 14.00 | 26.00 | 1.01 | 198.94 |
| totS | 69.00 | 4.25 | 62.00 | 78.00 | 1.01 | 274.36 |
